# The Possible Therapeutic Effects of Some Medicinal Plants for Chronic Cough in Children

**DOI:** 10.1155/2020/2149328

**Published:** 2020-10-01

**Authors:** S. Gholamreza Mortazavi Moghaddam, Majid Kianmehr, Mohammad Reza Khazdair

**Affiliations:** ^1^Cardiovascular Diseases Research Center, Birjand University of Medical Sciences, Birjand, Iran; ^2^Esfarayen Faculty of Medical Sciences, Esfarayen, Iran

## Abstract

The use of plants or their isolated bioactive components for the prevention and treatment of various disorders has been developed. Here, we aim to identify effective medicinal plants for relief of cough and respiratory symptoms in children. The data of this review article were obtained from published articles in scientific databases, including PubMed, Google Scholar, and Scopus, until December 2019. The keywords, including “*Zataria multiflora* Boiss.” OR “*Portulaca oleracea* L.” OR “*Ferula assa-foetida* L.” OR “*Nigella sativa* L.” AND “respiratory symptoms” OR “airway inflammation” OR “smooth muscle relaxant effects,” were searched individually or combined. The mentioned medicinal plants decreased total white blood cell (WBC), neutrophils, and eosinophils counts of blood and lung lavage in animal model-induced respiratory disorders. These plants also have protective effects on serum immunoglobulin, antibody titer, eosinophil count, and proinflammatory cytokines. Evidence from the studies indicated that the abovementioned medicinal plants have smooth muscle relaxant properties (bronchodilator effects) via stimulation of *β*-adrenoceptor or inhibition of muscarinic receptors (*in vitro*) and also improved the pulmonary function test in clinical settings. These medicinal plants are safe and easy to use. Based on the anti-inflammatory, anti-antispasmodic, and immunomodulatory effects, the clinical benefit may be assumed, therefore considering a place for these medicinal plants in relieve of chronic cough and symptoms of children's allergy, asthma, and common cold.

## 1. Introduction

Common cold is a kind of mild and self-limited respiratory illness caused by several different viruses [1]. Influenza virus is prevalent in winter, which affects a wide people in community. Influenza infection may lead to bacterial infections such as sinusitis, bronchitis, and pneumonia. People with declining immune systems or immune deficiency disorders and also children less than three years old make up high risk groups [2]. Cough is one of the most common symptoms of upper respiratory tract infection, specially in children [3]. It has been reported that a cough is defined as acute cough after <14-day duration, while definition for chronic cough varied from 3- to more than 12-week duration [4]. Cough in children causes significant anxiety to parents, and the use of incorrect or unnecessary drugs for cough treatment is related to adverse events [5]. Different class of drugs have been used for symptomatic treatment of cough, including antihistamines, decongestants, expectorants, mucolytics, and bronchodilators [6].

It should be noted that a significant number of antitussive drugs were obtained from natural products and plants in ancient times. Herbal medicines are popular in most of countries, and people who have used them possess valuable information about these plants [7, 8]. For this reason, the use of these drugs in outpatient treatments has been of great interest and so accepted by most of people. However, proper use of medicinal plants requires a lot of knowledge and experience, which has been unfortunately distorted over time [9, 10]. In Iranian traditional medicine, *Zataria multiflora* Boiss. was used for the treatment of various disorders, including remedy of coughs due to colds, oral cavity infection, dyspepsia, and also an antibacterial agent [11, 12]. *Portulaca oleracea* L. is a plant cold in nature and sour in taste, which is used to cool the blood and also used for treatment of fever, dysentery, diarrhea, eczema, and hematochezia in Chinese traditional medicine [13, 14].

Furthermore, this plant is used traditionally for gastrointestinal diseases, respiratory problems, and liver inflammation [15]. *Ferula assa-foetida* L. is usually used for the treatment of cough and pneumonia and gastrointestinal parasites and also as an anticoagulant [16, 17]. *Nigella sativa* seeds are used as a diuretic, liver tonic, and digestive. This plant is also used traditionally for the treatment of diarrhea, dyspnea, headache, influenza, and cough in the Middle East, India, and Pakistan [18].

This study aimed to search scientific database and identify the medicinal plants used as a spice and food additive in treatment of cold and cough.

## 2. Pharmacologic Mediations for the Treatment of Chronic Cough

### 2.1. Anti-Inflammatory Drugs

Combination of anti-inflammatory drugs and acetaminophen seem to be effective in relieving pain and fever in patients with upper respiratory tract infection [19].

### 2.2. Antihistamines Drugs

Antihistamines as monotherapy have no significant effect on the treatment of cold and cough [20]. Antihistamines, combined with decongestants, have a mild to moderate impact on common cold in older children and adults [21].

### 2.3. Antiviral Drugs

Among a large number of common cold viruses, rhinoviruses are the most common [22]. The antiviral drugs share a common mechanism of action, binding to specific hydrophobic pockets in virion capsid and inhibiting virion attachment or uncoating [23]. On the other hand, numerous studies have showed that different types of antiviral drugs have no effect against rhinovirus in clinical setting [24, 25].

### 2.4. Bronchodilator Drugs

Medications used to relieve acute respiratory symptoms induced by cold as well as asthma include short-acting bronchodilators [26], long-acting *β*-agonists [27], or the combination of long-acting *β*-agonists and inhaled corticosteroid [28, 29].

## 3. Results

### 3.1. Traditional and Phytomedicine Uses of Plants

Phytomedicine is defined as the use of plants or their isolated chemical components for the prevention and treatment of various disorders [30]. Plants or natural product agents were used since ancient times for treatment or control of various diseases; however, the use of herbal medicine has increased in recent decades. According to the World Health Organization reports, approximately 80% of the population of developing countries, currently, believed that phytomedicines are an affordable source of medication [31]. Plants contain numerous bioactive compounds in which many of them showed antimicrobial properties [32]. Plant-derived medicines have been part of folk healthcare in the most parts of the world for hundreds of years, and there is increasing interest in plants as sources of agents to fight microbial diseases in modern countries [33].

### 3.2. *Zataria multiflora* Boiss

*Zataria multiflora* Boiss. (*Z. multiflora*), which belongs to the Lamiaceae family, has been traditionally used as an anesthetic, antispasmodic [34], antiseptic, antidiarrheal, and analgesic agents [35]. The neuroprotective effect of *Z. multiflora* (used as a spice) was also reported [36]. Antioxidant, anti-inflammatory, and immunomodulatory properties of *Z. multiflora* and its constituents have already been reported [37]. This plant has also been used for treatment of dyspepsia and bloating [12] as well as vomiting, headache, migraine, premature labor pain, and common cold [38]. One study about essential oils extracted from *Z. multiflora* revealed its inhibitory effect on growth of yeasts and also antimicrobial activities against Gram-positive and negative bacteria at 0.12 to 8 *μ*L/mL concentrations [39].

#### 3.2.1. Anti-Inflammatory Effects

Therapeutic effects of *Z. multiflora* on inflammation in the lung and remedy of cough due to colds have been reported in traditional medicine [40]. The hydroethanolic extract of *Z. multiflora* decreased total white blood cell (WBC), neutrophils, and eosinophils counts in the animal model-induced chronic obstructive pulmonary disease (COPD) [41]. The beneficial effects of *Z. multiflora* extract on tracheal responsiveness and emphysema in the animals model of COPD have also been reported [42]. Moreover, there are reports about the protective effects of *Z. multi*ﬂ*ora* on total and differential WBC in bronchoalveolar lavage fluid (BALF) as well as on lung pathology, serum levels of phospholipase A2 (PLA2), total protein, and histamine of ovalbumin- (OVA-) sensitized animals [43]. What follows from these findings is that *Z. multiflora* has probably potential therapeutic effects on respiratory disorders associated with inflammation in the lung, such as asthma and COPD.

The aqueous and ethanolic extracts of *Z. multiflora* (0.08 and 1.4 g/kg, respectively) showed antinociceptive activities against xylene (0.03 ml) induced acute and acetic acid-induced (0.7%, i.p.) chronic inflammation [44]. Intraperitoneal administration of hydroalcoholic extract and essential oil of *Z. multiflora* (500 mg/kg, and 0.3 mL/kg, i.p., respectively) showed antinociceptive effects in the acetic acid-induced writhing test in rats [45]. It may be concluded that the extract of *Z. multiflora* has antinociceptive effects mediated by its anti-inflammatory property.

The methanolic extracts of *Z. multiflora* significantly decreased the macroscopic and microscopic scores in inflammatory bowel diseases (IBD) induced by intrarectal administration of acetic acid (0.1 ml) [46].

Pretreatment of LPS‐stimulated murine macrophages with essential oils extracted from *Z. multiflora,* including 52% carvacrol, 16% thymol, and 10% *p*‐cymene, leads to a significant reduction in H_2_O_2_ and nitric oxide (NO) production. This effect is mediated by inhibition of oxidative stress process or by radical scavenging activity of phenolic groups of the essential oil such as thymol and carvacrol. Therefore, *Z. multiflora* extract or its essential oil can be used in the therapy of oxidative damage induced by inflammation [47]. The findings prove that *Z. multiflora* has potential of reduction in inflammation by reduce in H_2_O_2_, NO, differential WBC, and phospholipase A2 production.

#### 3.2.2. Relaxant Effect on Tracheal Smooth Muscle

The therapeutic effect of *Z. multiflora* in respiratory disorders of chemical war victims [48] and its antitussive effect were suggested, which could be due to its relaxant effect on airway smooth muscle leading to bronchodilation [49].

Long-term administration of *Z. multiflora* hydroethanolic extract also caused a reduction in tracheobronchial hyperresponsiveness in animal model of asthma [43] and COPD [42]. The possible antagonistic effect of the hydroethanolic extract of *Z. multiflora* on muscarinic receptors of incubated tracheal chains with propranolol and chlorpheniramine versus to those of nonincubated tissue was investigated [50].

The inhibitory effects of *Z. multiflora* on histamine (H1) receptors of the incubated trachea with 1.4 *μ*M indomethacin and 1 *μ*M propranolol versus to those of nonincubated tissue were reported [51]. In a similar study, *Z. multiflora* extract (2.5, 5, and 10 *μ*g/mL) and carvacrol (1, 2, and 4 *μ*g/mL) showed also inhibitory effect on histamine (H_1_) receptors [52]. The stimulatory effects of *Z. multiflora* on *β*2-adrenoceptor receptors of incubated guinea pig tracheal smooth muscle with isoprenaline versus to those of nonincubated tissue were reported [53].

The inhibitory effects of *Z. multiflora* extract on voltage-dependent calcium channels of the ileum smooth muscle were also reported [54, 55].

KCL but not acetylcholine induced uterus muscle contraction was reversed by *Z. multiﬂora* extract (2 mg/ml) which is more potent than the atropine effect [54]. Because KCl induced muscle contraction is mediated by voltage-dependent calcium channel, these findings support a calcium channel blocking effect for the *Z. multiﬂora* extract.

Carvacrol has an inhibitory effect on L-NAME induced hypertension mediated by cardiac L-type calcium channel blocking action [56].

In conclusion of the referred studies, *Z. multiﬂora* reduces inflammation in the respiratory system and also declines smooth muscle contraction by action on various tracheal smooth muscle receptors, which lead to a relaxant effect. Therefore, the use of this plant may be useful for obstructive airway disorders, including asthma and COPD. The anti-inflammatory and smooth muscle relaxant effects of *Z. Multiflora* are summarized in Table 1.

#### 3.2.3. Clinical Evidences

*Z. multiflora* and its main constituent, carvacrol, reduce inflammatory and oxidant markers and increase antioxidant markers and improve pulmonary function tests (PFT) in patients who were exposed to sulfur mustard in past (SM) and suffered from lung injuries [57, 58]. *Z. multiflora* syrup (5 and 10 mg/kg/day) and carvacrol (1.2 mg/kg/day) significantly reduce chest wheeze in asthmatic patients. In addition, *Z. multiflora* and carvacrol both significantly increase forced expiratory volume in 1 second (FEV_1_%) and decrease the plasma level of NO^2−^ after two months of treatment [59].

Two-month treatment with *Z. multiflora* extracts (5 and 10 mg/kg/day) significantly decreases serum levels of inflammatory cytokines, including IL-2, IL-6, and IL-8; it also significantly raises serum levels of IL-10 and IFN-*γ* in patients exposed to SM. Furthermore, *Z. multiflora* increases maximum midexpiratory flow (MMEF) and maximum expiratory flow at 25, 50, and 75% of vital capacity (VC) (MEF25, 50, and 75) of treatment [60].

A similar study showed that *Z. multiflora* was able to significantly lower serum levels of tumor necrosis factor (TNF-*α*), monocyte chemotactic protein 1 (MCP-1), vascular endothelial growth factor (VEGF), and epidermal growth factor (EGF) and also improve PFT test values and respiratory symptoms in the SM exposed patients [61]. In addition, *Z. multiflora* (5 and 10 mg/kg/day) significantly improves clinical symptoms, PFTs values, oxidative stress, and cytokine levels in asthmatic patients two months after treatment compared to baseline [62]. Taken together, these studies confirm the efficacy of *Z. multiflora* for prevention and treatment of respiratory disease in adults and children.

### 3.3. *Portulaca oleracea* L

*Portulaca oleracea* L. (*P. oleracea*), or Purslane from Portulacaceae family, is an annual and grassy plant with small yellow flowers, which grows in different parts of the world including Europe, Indies, China, Japan, and Iran [63]. *P. oleracea* contains omega 3 fatty acids and alpha-linolenic acid more than other leafy vegetable plants [64]. Antitussive [65], analgesic, and anti-inflammatory [66], as well as the neuroprotective, effect of *P. oleracea* were reported [36]. The ethanol extracts of *P. oleracea* (40 mg/mL) showed antimicrobial properties against five bacteria and three fungi by agar diffusion method [67].

#### 3.3.1. Anti-Inflammatory Effects

The hydroethanolic extracts (10, 40, and 160 *μ*g/ml) of *P. oleracea* significantly increases anti-inflammatory cytokine (IFN-*γ*) in nonstimulated and stimulated human lymphocytes cells. In stimulated lymphocytes, the extract of *P. oleracea* significantly decreases inflammatory cytokines such as IL-4, IL-10, and free radicals such as NO [68]. The aqueous extracts of *P. oleracea* (10, 25, 50, and 100 *μ*g/ml) in a dose-dependent manner significantly inhibit TNF-*α*-induced intracellular reactive oxygen species (ROS) production. *P. oleracea* also suppressed the TNF-*α*-induced degradation of I*κ*B-*α* and reduces the TNF-*α*-induced NF-*κ*B binding protein in the vascular endothelial cells. The plant extracts also effectively reduce the mRNA expression of monocyte chemoattractant protein 1 (MCP-1) and IL-8 induced by TNF-*α* [69]. The ethanol extract of *P. oleracea* inhibited the production of inflammatory mediators such as NO and proinflammatory cytokines, including TNF-*α*, IL-1*β*, and IL-6 in LPS-induced inflammation in RAW 264.7 cells (derived from BALB/c mice). *P. oleracea* extracts also inhibited the phosphorylation of (ERK1/2), c-Jun NH_2_-terminal kinase (JNK), and NF-*κ*B activation in cells [70]. POL-P3b as a polysaccharide fraction purified extracted from *P. oleracea* upregulates the expression of CD80, CD86, and CD83 and stimulates production of IL-12 and TNF-*α* in large quantities and IL-10 in small quantities by improving the maturation and function of murine bone-marrow-derived dendritic cells (DCs). Furthermore, POL-P3b significantly increased the expression of Toll-like receptor 4 (TLR-4) on DCs treated. The findings confirm that POL-P3b is able to induce DCs maturation through TLR-4 [71]. The oral administration of *P. oleracea* polysaccharides significantly and dose-dependently increases stimulation indices (SI) of T lymphocytes and B lymphocytes in Wistar rats [72]. The polysaccharide (POP) from *P. oleracea* shows the preventive effect on the reduction of the spleen weight and the number of murine spleen T cells after 30 days of inducing age in the mice with D-galactose [73]. *P. oleracea* (50–200 mg/kg) significantly reduces IL-*β*, IL-6, TNF-*α*, PGE_2_, and TGF-*β* and increases IL-10 levels in the bronchoalveolar lavage fluid (BALF) of lung treated by lipopolysaccharide- (LPS-) induced inflammation [74].

#### 3.3.2. Relaxant Effect on Tracheal Smooth Muscle

The relaxant effect of *P. oleracea* on skeletal muscle [75] and smooth muscle [76] has been shown. The hydroethanolic extract of *P. oleracea* showed a stimulatory effect on *β*-adrenoceptor in the tracheal smooth muscles of guinea pigs [77]. The relaxant effect of *P. oleracea* on tracheal smooth muscles via the blocking of the muscarinic receptor was also investigated [78]. The researchers suggested that bronchodilatory effects of *P. oleracea* can be attributed to a variety of mechanisms, including *β*_2_-adrenoceptors stimulation [79], strengthening the inhibitory effect of nonadrenergic and noncholinergic nervous system [76], opening potassium channels [80], inhibition of phosphodiesterase [81], and calcium channel antagonism in tracheal chain [82]. Ethyl acetate (EA) of *P. oleracea* extract has been indicated to decrease intestinal motility in ICR mice compared to those treated with acetylcholine [83]. Based on the conducted studies, the aqueous extract of *P. oleracea* has been shown to enhance acetylcholine (Ach) and sodium nitroprusside (SNP) induced vascular relaxation of aortic rings in diabetic mice. The created effect is associated with a significant reduction in the level of vasoconstrictor endothelin- (ET-) 1. It has also been suggested that the aqueous extract of *P. oleracea* suppresses overexpression of vascular cell adhesion molecule- (VCAM) 1, intracellular cell adhesion molecular- (ICAM) 1, E-selectin, and matrix metalloproteinase (MMP) 2 in aortic tissue in db/db mice [84].

Aqueous extract of *P. oleracea* reduced the peristaltic index by antagonistic effects on calcium channel in the isolated guinea pig ileum strip [85]. The aqueous extract of the plant also showed a relaxant effect on smooth muscle of vasculatures, guinea pig fundus, rabbit jejunum, and rabbit aorta [86]. Therapeutic effect of *P. oleracea* in the airway of asthmatic patients showed that the oral admonition of 5% boiled extract (0.25 ml/kg) improved pulmonary function tests similar to theophylline [76].

Based on the referred studies, the *P. oleracea* has inhibitory effect on smooth muscle contraction, cytokine production, and inflammation in the respiratory system that may be useful for obstructive airway disorders, including asthma and COPD. The anti-inflammatory and smooth muscle relaxant effects of *P. oleracea* are presented in Table 2.

#### 3.3.3. Clinical Evidences

Bronchodilatory effect of the boiled extract of *P. oleracea* in asthmatic patients was revealed by enhanced all measured PFTs. It has also been reported that this bronchodilatory effect is equivalent to theophylline syrup [76]. Taking *P. oleracea* seeds (5 g) twice a day significantly decreases serum levels of lipid profiles and fasting blood glucose in subject with type 2 diabetes [87].

Topical administration of *P. oleracea* aqueous extract (140 mglml) in patient with incomplete injury of the spinal cord (T6) significantly reduced muscle spasm more than 50%. Reductions in tone were recorded in some patients with flexor or extensor contractures [88].

Daily supplement of *P. oleracea* (7.5 grams) for 8 weeks in type 2 diabetic women significantly reduced matrix metalloproteinases 2 and 9 (MMP2 and MMP9) and tissue inhibitor of matrix metalloproteinase (TIMP1) [89].

### 3.4. *Ferula assa-foetida* L

*Ferula assa-foetida* L. (*F. assa-foetida* L.) or asafoetida belongs to the Apiaceae family. Its gum resin is obtained from the exudates of the living underground rhizome or taproots of the plant. *F. assa-foetida* or gum-resin is known as “Anghouzeh,” “Khorakoma,” and “Anguzakoma” in Iran [90]. One conducted study revealed that the sesquiterpene coumarins, isolated from CHCl3-soluble extract of *F. assa-foetida*, showed higher potency against the influenza A virus (H1N1) (IC50 0.26–0.86 *μ*g/mL) than amantadine (IC50 0.92 *μ*g/mL) [91].

#### 3.4.1. Anti-Inflammatory Effects of *F. assa-foetida*

The roots, young shoots, and leaves of *F. assa-foetida* are eaten as a vegetable. Leaves of the plant possess anthelmintic, carminative, and diaphoretic properties and the root of the plant are used as antipyretic [92]. *F. assa-foetida* is also used for the treatment of various inflammatory diseases, including asthma, stomachache, flatulence, intestinal parasites, poor digestion, and influenza in traditional medicine [91]. Furthermore, previous recent study also reported that oleogum resin of *F. assa-foetida* possesses sedative, expectorant, analgesic, antidiabetic, antispasmodic, anti-inflammatory, and antiepileptic effects [93]. The oleogum resin of *F. assa-foetida* also showed antioxidant, antiviral, antifungal, antispasmodic, and antihypertensive effects in pharmacological studies [90]. It has been documented that *F. assa-foetida* resin can potentially inhibit monoamine oxidase B (MAO-B), and it can be used in the therapy of neurodegenerative diseases such as Parkinson's and Alzheimer's diseases [94].

#### 3.4.2. Relaxant Effect on Tracheal Smooth Muscle

The relaxant effect of aqueous extract of *F. assa-foetida* (2, 5, and 10 mg/ml) on the smooth muscle of the guinea pig tracheal chain (*in vitro* study) was reported [95]. The relaxant effect of *F. assa-foetida* extract via inhibitory effect on histamine (H1) receptors, muscarinic receptors, and possible mechanisms for functional antagonistic on tracheal smooth muscle) have been investigated and [96, 97] also reviewed [98].

The inhibitory effect of ethanolic root extract of *the Ferula* genus (*Ferula sinaica*) on rabbit tracheal contraction induced by acetylcholine and also guinea pig uterine smooth muscle contractions induced by oxytocin have been reported [99]. In a similar study, *Ferula sinaica* (50 mg) inhibited the histamine (10^−4^ M) induced contractions of guinea pig tracheal smooth muscle [100].

The relaxant effects of *F. assa-foetida* and its main constituents (umbelliprenin) on the tracheal smooth muscle contracted by methacholine and KCL were also reported [101]. It has been reported that *F. assa-foetida* extract (1–7 mg/ml) reduces the spontaneous contraction of the isolated guinea-pig ileum and also contraction induced by KCl (28 mM), acetylcholine (20 *μ*M), and histamine (20 *μ*M). Extract of the plant (3 mg/ml) has antagonists property in guinea-pig isolated ileum precontracted with KCl and also has cyclooxygenase inhibitor property [102]. *F. assa-foetida* seed's essential oil (0.1, 0.2, and 0.3%) showed antispasmodic action on isolated rat's ileum contraction induced by acetylcholine [103]. *F. assa-foetida* extract (2.2 mg/100 g, b.w.) significantly reduces the mean arterial blood pressure in anesthetized rats [102]. In one study it was shown that *F. sinaica* root extract (50 mg) has inhibitory effects on rabbit aorta contractions induced by norepinephrine (10^−4^ M) [100]. Moreover, the vasodilatation property of *F. assa-foetida* extracts (180 and 360 mg/ml) on arterial rings was suggested [104].

The anti-inflammatory and smooth muscle relaxant effects of *F. assa-foetida* are reported in Table 3.

#### 3.4.3. Clinical Evidences

The safety and efficacy of *F. assa-foetida* on treatment of functional dyspepsia (FD) in a double-blinded, placebo-controlled study showed that treatment with *F. assa-foetida* (250 mg × 2/day) (*n* = 21) for 30 days significantly improved the overall score and quality of life compared to the placebo group. In addition, treatment with *F. assa-foetida* eliminates and improve the symptoms, including bloating (58%), appetite (69%), postprandial fullness (74%), motion sickness (75%), and indigestion (77%) as compared to less than 10% improvement of symptoms in the placebo group [105]. The effects of *F. assa-foetida* mouthwash (10%) twice daily for a period of 7 days compared to chlorhexidine gluconate (CHG) mouthwash (15 ml) were also studied. The result of the research was that modified gingival index (MGI) and the plaque index (PI) were improved in both groups of intervention. However, mean differences of MGI and PI in *F. assa-foetida* group were lower than the CHG group [106]. The effects of 50% water-ethanol roots extracts of *F. assa-foetida* prepared as masculine tablet (310 mg) on young men for 3 months were studied. Masculinity reduced production and release of MDA in human sperm cells. Furthermore, treatment with masculine tablet increased sperm motility and sperm count in 15 oligospermic volunteers (60%) compared with the baseline of the study [104].

### 3.5. *Nigella sativa* L

*Nigella sativa* L. (*N. sativa*) or black seed is an annual plant that belongs to the Ranunculaceae family. It grows natively in Southwest Asia, Southern Europe, and North Africa and cultivates in different parts of the world [107]. *N. sativa* has been used in folk medicine for treatment of fever, infection, inflammation, chest congestion, cough, bronchitis, asthma, dysmenorrhea, diabetes, flatulence, dyspepsia, diarrhea, and dysentery [108, 109]. Pharmacological effects of *N. sativa* and its active constituent, thymoquinone (TQ), including anti-inflammatory, antioxidant, neuroprotective, and renoprotective effects has been reported [110–113]. *N. sativa* extract also showed the highest antibacterial and inhibitory activity against zucchini yellow mosaic virus (ZYMV) [114].

#### 3.5.1. Anti-Inflammatory Effects of *N. sativa*

Culture medium of nonactivated peripheral blood mononuclear cell (PBMC) and allogeneic cells exposed to *N. sativa* extract (1 and 2 *μ*g/ml) stimulate production of IL-1*β* and IL-4 levels. Furthermore, *N. sativa* (10 *μ*g/ml) suppresses the production of IL-8 in nonstimulated as well as mitogen-activated PBMC cells [115]. *N. sativa* aqueous extract (50 *μ*g/ml) suppresses lymphocytes response to all mitogens and allogeneic cells. However, *N. sativa* (0.5 *μ*g/ml) stimulates lymphocytes response to allogeneic cells. Moreover, the fraction of *N. sativa* (10 kDa) stimulates the production of IL-1*β* and IL-3 by human lymphocytes without need for any kind of mitogen. *N. sativa* (0.5 *μ*g/ml) also significantly increased IL-3 production when it was added to lymphocytes culture [116].

The preventive effects of *N. sativa* extract (1.25 and 2.50 g/L, p.o.) on guinea pigs OVA-induced asthma significantly decreased the levels of IL-4 and pathological changes, including intra-alveolar hemorrhage and inflammatory cells of the lung, but increased IFN-*γ* [117]. In another experiment, the hydroethanolic extract of *N. sativa* (0.08 g in drinking water) decreased neutrophil numbers and restored IL-4, and IFN-*γ* levels in sulfur mustard (40 mg/m^3^) exposed animals [117, 118].

Intragastric gavage of *N. sativa* oil (5 ml/kg) showed anti-inflammatory effects on conalbumin-induced asthma in mice. Peripheral blood eosinophil count, IgG1 and IgG2a levels, and cytokines, including IL-2, IL-12, and IFN-*γ* levels in lung tissue, were significantly decreased [119].

Administration of *N. sativa* oil (5 ml/kg, intragastrically) in mice model of allergic asthma significantly reduced peripheral blood eosinophils and lung inflammation but did not reduce lung tissue induced nitric oxide synthase (iNOS) expression compared with the control group [120].

Treatment of OVA-sensitized mice with the main component of *N. sativa*, TQ (3 mg/kg, i.p.) for five days period definitely decreased sensitivity of the tracheal smooth muscle aroused by acetylcholine and histamine 71% and 74%, respectively, compared to the sensitized animals. TQ (8 mg/kg, i.p.) prevented most of the pathological changes due to lipopolysaccharide- (LPS-) induced inflammatory cells infiltration, lipid peroxidation (LP), glutathione depletion (GSH), TNF-*α,* and IL-1*β* levels in both BALF and lung tissue homogenates [121].

Intraperitoneal administration of TQ (3 mg/kg) before OVA-sensitized mice airway significantly decreased eosinophil count in the lung and also increased Th2 cytokines in the BAL fluid after airway challenge with OVA antigen. TQ also reduced the serum levels of immunoglobulin (Ig) E and IgG1. Additionally, TQ significantly inhibited eosinophilic lung inflammation and mucus-producing goblet cells in the histological assessment. TQ also significantly reduces the levels of IL-4, IL-5, and IL-13 in the BAL fluid [122].

Administration of TQ (20 and 40 mg/kg, i.p.) and codeine (5 mg/kg), as a prototype antitussive agent, reduced the amount of cough in guinea pigs exposed to citric acid 20%. The antitussive effect of TQ was antagonized by pretreatment with an opioid receptor antagonist (naloxone, 2 mg/kg) [123].

The hydroethanolic extract of *N. sativa* (0.08 g daily) on sulfur mustard exposed guinea pigs caused the reduction of tracheal responsiveness to methacholine as well as total and differential WBC count in treated compared to untreated animals [124]. Similarly, *N. sativa* significantly decreased tracheal responsiveness and lung inflammation including the percentage of eosinophil and monocyte, neutrophil, and lymphocyte number in guinea pigs 14 days after sulfur mustard exposure [118].

Coadministration of *N. sativa* seeds (2 g) with bee honey (1 teaspoon per day), for three month-duration, in asthmatics and nonasthmatics subjects (8 to 40 years) in Khartoum showed significantly improvement in forced vital capacity (FVC) in asthmatics' group and peak expiratory flow rate (PEFR) in nonasthmatics' group [125].

Administration of *N. sativa* capsules (40–80 mg/kg/day, p.o.) in adults and children with allergic rhinitis, atopic eczema, and asthma significantly reduced plasma and urine levels of IgE, eosinophil count, and endogenous cortisol compared to their pretreatment values [126].

The effect of *N. sativa* (1 and 2 g/day for 6- to 12-week duration) supplementation decreased inflammation of the airways and also reversed limitation of airflow, including forced midexpiratory flow (FEF) 25–75% and forced expiratory volume in 1 second (FEV1) % in asthma patients. In addition, *N. sativa* (1 and 2 g/day) significantly improved PEF variability after 6 and 12 weeks of treatment compared to the controls group. The plant also decreased fractional exhaled nitric oxide (FeNO) and serum IgE after 12 weeks of treatment compared to the baseline. *N. Sativa* also increased serum IFN-gamma as well as improved in the asthma control test (ACT) score [127].

The finding from different studies indicated that *N. sativa* has some effects on serum immunoglobulin, antibody titer, eosinophil count, and cytokine profiles.

#### 3.5.2. Relaxant Effect on Tracheal Smooth Muscle

The crude extract of *N. sativa* seeds (0.1–3.0 mg/ml) exhibited contraction effect on rabbit jejunum. The extract of the plant creates a dose-dependent shift to the right in the Ca^2+^ dose-response curves similar to verapamil and exhibits a calcium channel blocker effect. *N. sativa* extract (0.1–3.0 mg/ml) also has a relaxant effect on carbachol, histamine, or K(+) induced contractions in guinea pig trachea smooth muscle trough calcium channel blocking effects [128]. The aqueous extract of *N. sativa* (0.25, 0.5, and 1 g%) creates the rightward shift in calcium concentration-response curves of calcium-induced contraction of guinea pig tracheal smooth muscle, which may be due to calcium antagonist effects of the extract [129].

The relaxant effect of the aqueous extract of *N. sativa* (0.25, 0.5, and 1 g %) on the guinea pig's isolated trachea chain was also examined. The extract of *N. sativa* compared with saline showed a dose-dependent stimulatory effect on *β*2-adrenoceptors and eventually led to tracheal smooth muscles relaxation [130]. The aqueous fractions of *N. sativa* (0.8, 1.2, 1.6, and 2.0 g%) showed significant relaxant effects on precontracted guinea pig tracheal chains by KCl (60 mM) and methacholine (10 *μ*M) [131].

The hydroethanolic extract of *N. sativa* (0.125 and 0.250 mg/ml) significantly decreased tracheal response of isolated trachea chain to methacholine and ovalbumin compared to the controls. Moreover, the extract significantly decreased WBC cell and eosinophil counts in the lung lavage fluid [132].

In another study, the relaxant effects of methanolic fractions (20%, 40%, 60%, 80%, and 100%) of *N. sativa* on precontracted tracheal smooth muscle of guinea pig by KCl and methacholine demonstrated significant relaxant effects to the extent that 20% methanolic fraction had higher relaxant effect than theophylline [133].

The vasodilator effect of *N. sativa* extract (2–14 mg/mL) on KCl induced contractile responses of the isolated aorta and also on endothelium-intact and endothelium-denuded aortic rings precontracted by phenylephrine and KCl has seen [134]. The bronchodilatory effects of *N. sativa* boiled extract (50 and 100 mg/kg) compared to theophylline (6 mg/kg) in asthmatic patients (*n* = 15) become obvious in improvement of all measured PFTs values, in most time intervals. However, the increase in PFT values was significantly lower than those of theophylline, whereas the onset of the bronchodilatory effect of the extract was similar to that of theophylline beginning 30 min after administration [135].

The results of these studies indicated that *N. sativa* could be applied for the treatment of inﬂammatory disorders, including allergy and asthma. Noteworthy, anti-inflammatory effects of the plant indicated that these plants and their main components might be useful for treatments of respiratory disorders such as cold and improves some measures of pulmonary function and subsides respiratory symptoms such as cough in children and adults. The anti-inflammatory and smooth muscle relaxant effects of *N. sativa* are shown in Table 4.

#### 3.5.3. Clinical Evidences

Treatment of rheumatoid arthritis (RA) patients with two capsules of *Nigella sativa* oil (500 mg, per day for 8 weeks) significantly increased IL-10 serum level, whereas it decreased serum levels of MDA and nitric oxide compared with the placebo group [136].

Whole ground seeds of *N. sativa* (1 and 2 g/day, capsules) significantly decreased fractional exhaled nitric oxide (FeNO) and serum immunoglobulin E (IgE) in asthmatic patients after 12 weeks compared to the baseline. Serum levels of IFN-*γ* were significantly increased after 12 weeks of treatment with *N. sativa* compared to the baseline [127]. Treatment with soft gel capsules of *N. sativa* oil (15–30 mg/kg/day) significantly increased serum levels of IFN-*γ* and reduced serum levels of IL-4 in asthmatic children (aged 6–15) compared to control [137]. In one study, researchers showed that the oils of *N. sativa* (15–30 mg/kg) significantly decreased Th17 and also increased Treg percentages. In the same way, Th17/Treg ratio was lower in *N. sativa* treated group compared to the standard treated group [138]. Supplementation with *N. sativa* oil in patients with nonalcoholic fatty liver disease (NAFLD) for 8 weeks decreased the level of FBS, lipid profiles (TG, TC, LDL, and VLDL), liver enzymes (AST and ALT), inflammatory marker (hs-CRP, IL-6, and TNF-*α*), whereas it increased the HDL-C levels compared to the placebo (paraffin oil) group [139]. *N. sativa* supplementation (2 g/day for 12 weeks) in patients who have cardiovascular disorders risk factors and also in patients with NAFLD 2 significantly decreased glucose and insulin serum levels and also decreased hepatic steatosis percentage compared to the placebo [140].

**Figure 1:**
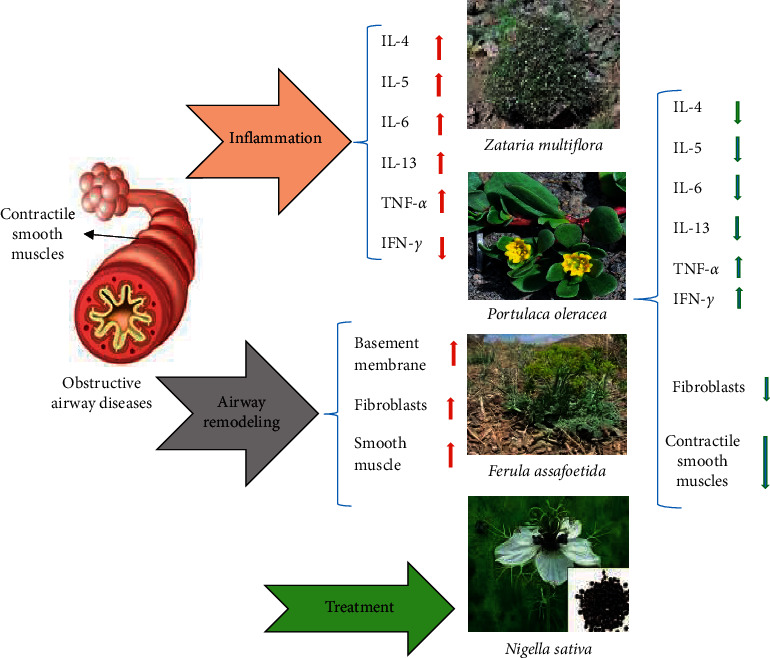
The possible therapeutic actions of the plants on the respiratory system.

Potential therapeutic effects of the medicinal plants on respiratory system, including anti-inflammatory and relaxant effects on tracheal smooth muscle, are shown in Figure 1.

## 4. Conclusion

This review highlighted the anti-inflammatory and smooth muscle relaxant effects of various medicinal plants that have been used frequently for dietary, food additive, and spice among the people of different countries. They have anti-inflammatory properties, including reduction in inflammatory cytokine, total white blood cells, neutrophils, and eosinophils in the blood and BAL fluid in induced asthma or COPD of animal's model. Furthermore, these plants attenuated tracheal responsiveness and smooth muscle contraction via inhibition of histamine and muscarinic receptors or via agonistic effects on *β* adrenergic receptors. Therefore, according to the basic and clinical evidence, these plants have potential of therapeutic effects on allergic asthma and obstructive airway disorders. So, because of safety and easy use, these medicinal plants and their main components can be suggested for treatments of acute cough in patients with cold and also for relief of chronic cough in patients with chronic respiratory disorders such as COPD and especially in childhood asthma.

## Figures and Tables

**Table 1:** Anti-inflammatory and smooth muscle relaxant effects of *Z. multiflora*.

Extract	Doses	Model of study	Effects	Reference
Aqueous ethanolic	0.4, 0.8, and 1.6 mg/ml, p.o.	Guinea pigs model of COPD	Improved the levels of IL-8, total WBC number, and lymphocytes percentage	[41]
	0.4, 0.8 and 1.6 mg/ml	Guinea pigs model of COPD	Improved tracheal responsiveness (TR) and emphysema	[42]
	0.2, 0.4, and 0.8 mg/ml, p.o.	Guinea pigs model of asthma	Reduced tracheal responsiveness to methacholine and OVA, serum levels of NO, nitrite, and PLA2	[43]
	0.2, 0.8 and 1.4 g/kg, i.p.	Mice model of paw inflammation	Inhibited acute inflammation	[44]
	0.2, 0.8 and 1.4 g/kg, i.p.	Rat model of paw inflammation	Inhibited chronic inflammation	[44]
Aqueous ethanolic and essential oil	500 and 900 and essential oil 0.3 ml/kg, i.p.	Tail flick test	Showed anti-inflammatory effect	[45]
Aqueous methanolic	400, 600, 900 p.p.m. in drinking water	Albino mice model of bowel inflammation	Reduced score of macroscopic and microscopic characters acetic acid-treated group	[46]
Aqueous ethanolic	5 and 10 mg/kg	SM exposed patients	Reduced inflammatory and oxidant markers, but improved PFT values	[57, 58]
Aqueous ethanolic	Unknown	SM exposed patients	Showed bronchodilatory effects	[48]
Hydroethanolic	0.2, 0.4 and 0.8 mg/ml	Sensitized Guinea pigs	Reduced tracheal responsiveness	[43]
Hydroethanolic	(0.4, 0.8 and 1.6 mg/ml)	Guinea pigs model of COPD	Relaxed tracheal smooth muscle	[42]
Hydroethanolic	2.5, 5, and 10 *μ*g/mL	Tracheal chains	Showed antagonistic effects on muscarinic receptors	[50]
Hydroethanolic	2.5, 5, and 10 *μ*g/mL	Tracheal chains	Showed inhibitory effects on histamine (H1) receptors	[51]
Hydroethanolic	2.5, 5, and 10 *μ*g/mL	Tracheal chains	Showed stimulatory effects on *β*2-adrenoceptor receptors	[53]
Hydroalcoholic extract	1 and 2 mg/ml	Ileum smooth muscle	Showed inhibitory effect on voltage dependent calcium channels	[54]
Hydroalcoholic extract	2 mg/ml	Uterus muscle	Showed calcium channels blocking effect	[54]

TR, tracheal responsiveness; NO, nitric oxide; IL-1*β*, interleukin-1*β*; BAL, bronchoalveolar lavage; PFT, pulmonary function tests; SM, sulfur mustard; COPD, chronic obstructive pulmonary disease.

**Table 2:** Anti-inflammatory and smooth muscle relaxant effects of *P. oleracea*.

Extract	Doses	Model of study	Effects	Reference
Hydroethanolic	160 *μ*g/ml	Lymphocyte	Increased IL-4, IL-10, IFN-*γ*, IFN-*γ*/il-4, and IL-10/il-4 ratios	[68]
Aqueous extract	100 *μ*g/ml	Vascular endothelial cells	Decreased mRNA expressions of MCP-1 and IL-8	[69]
Ethanol	200 *μ*g/ml	RAW 264.7 cells	Decreased TNF-*α*, IL-1*β,* and IL-6	[70]
POL-P3b	(250 *μ*g/ml)	DCs	Increased IL-12 and IL-10	[71]
Polysaccharide	600 *μ*g/ml, p.o.	Rat	Increased T lymphocytes and B lymphocytes	[72]
Polysaccharide	Unknown	Mice	Showed preventive effect on reduction of the spleen weight and the number of murine spleen T cells	[73]
Boiled extract	0.25 mg/kg	Asthmatic patients	Increased measured PFTs	[76]
Aqueous extracts	0.06, 0.12 and 0.25 mg/ml	Tracheal chains of guinea pig	Showed stimulatory effect on *β*-adrenoceptor	[77]
Hydroethanolic	0.25, 0.50 and 1.00 mg/ml	Guinea pigs tracheal smooth muscles	Showed blocking effects of muscarinic receptor	[78]
Ethanolic extract	(250 *μ*g/ml)	Mice	Reduced intestinal motility	[83]
Aqueous extract	(300 mg/kg/day, po)	Mice aortic tissue	Suppressed overexpression of (VCAM) -1, (ICAM) - 1, E-selectin and (MMP) -2	[84]
Aqueous extract	600 *μ*g/ml p.o.	Isolated guinea pig ileum strip	Reduced peristaltic index	[85]
Aqueous extract	7 × 10^−4^ g/ml	Guinea pig fundus, rabbit jejunum and rabbit aorta	Showed relaxant effect on smooth muscle and reduced blood pressure	[86]

IL: interleukin, IFN-*γ*: interferon-gamma, POL-P3b: polysaccharide fraction, MCP-1: monocyte chemoattractant protein 1, TNF-*α*: tumor necrosis factor-*α*, NO: nitrogen oxide, IgE: immunoglobulin E, (VCAM) 1: vascular cell adhesion molecule, (ICAM) 1: intracellular cell adhesion molecular, and (MMP) 2: matrix metalloproteinase.

**Table 3:** Anti-inflammatory and smooth muscle relaxant effect of *F. assa-foetida*.

Extract	Doses	Model of study	Effects	Reference
Root extract	Unknown	Traditional medicine	Used for treatment of various inflammatory diseases including; asthma, stomachache, flatulence, intestinal parasites, weak digestion, and influenza.	[91]
Oleo gum resin	50 and 100 mg/kg	Seizures induced rat	Showed antispasmodic, anti-inflammatory, and antiepileptic effects.	[93]
Oleo gum resin	Unknown	Traditional uses	Showed antioxidant, antiviral, antifungal, antispasmodic, and antihypertensive effects.	[90]
Aqueous extract	2, 5 and 10 mg/ml	Guinea pigs tracheal smooth muscles	Showed relaxant effect on tracheal smooth muscle	[95]
Aqueous extract	2, 5 and 10 mg/ml	Guinea pigs tracheal smooth muscles	Showed competitive antagonistic effect at muscarinic receptors and inhibitory effect on histamine (H1) receptors of tracheal smooth muscles	[96, 97]
Root ethanolic extract	50–500 mg	Rat and guinea pig	Inhibited the contractions of rabbit tracheal induced by acetylcholine and contractions of guinea pig uterine smooth muscle induced by oxytocin	[99]
Aqueous extract	2, 5 and 10 mg/ml	Guinea pigs tracheal smooth muscles	Showed potent relaxant effect on tracheal smooth muscle	[101]
Aqueous extract	1, 2, 3, 5 and 7 mg/ml	Isolated guinea pig ileum	Reduced the spontaneous contraction of the isolated guinea-pig ileum	[102]
Seed's essential oil	0.1, 0.2 and 0.3%	Isolated rat's ileum	Showed antispasmodic effect on rat's ileum	[103]
Aqueous extract	2.2 mg/100 g, b.w	Rat	Reduced the mean arterial blood pressure	[102]
Root extract	50 mg	Rabbit aorta	Showed inhibitory effects on contractions of rabbit aorta induced by norepinephrine	[100]
Aqueous extract	(180 and 360 mg/ml)	Rat arterial rings	Showed vasodilatation effect	[104]

**Table 4:** Anti-inflammatory and smooth muscle relaxant effects of *N. sativa*.

Extract	Doses	Model of study	Effects	Reference
Aqueous ethanolic	10 *μ*g/ml, 1 and 2 *μ*g/ml	Culture medium of PBMC	Increased IL-1*β* and IL-4 levels but suppressed the production of IL-8	[115]
Aqueous	50 *μ*g/ml, 0.5 *μ*g/ml	Human lymphocytes culture	Suppressed lymphocytes response to all mitogens and allogeneic cells, Stimulated lymphocytes response to allogeneic cells, but stimulated the production of IL-1*β* and IL-3 and increased IL-3 production	[116]
Aqueous	1.25 and 2.50 g/L, p.o	Guinea pigs model of asthma	Decreased the levels of IL-4 and pathological changes, while increased IFN-*γ*	[117]
Aqueous	0.08 g in drinking water	Sulfur mustard exposed Guinea pigs	Decreased neutrophil numbers and restored IL-4 and IFN-*γ* levels	[117, 118]
*N. sativa* oil	5 ml/kg	Mice model of asthma	Decreased Peripheral blood eosinophil count, IgG1 and IgG2a levels, and cytokines including; IL-2, IL-12, and IFN-*γ* levels in lung tissue	[119]
*N. sativa* oil	5 ml/kg intragastrically	Mice model of asthma	Reduced peripheral blood eosinophils and lung inflammation	[120]
Main component, TQ	3 mg/kg, 8 mg/kg i.p.	OVA sensitization mice	Decreased sensitivity of the tracheal smooth muscle by acetylcholine and histamine, cells infiltration, TNF- *α,* and IL-1*β* levels in both bronchoalveolar lavage fluid (BALF) and lung tissue homogenates	[121]
Main component, TQ	3 mg/kg, i.p.	OVA sensitization mice	Decreased eosinophil count in the lung and increased Th2 cytokines in the BAL fluid, reduced the elevated serum levels of immunoglobulin (Ig)-E and IgG1, inhibited lung eosinophilic inflammation and mucus-producing goblet cells, inhibited the levels of IL-4, IL-5 and IL-13 in the BAL fluid	[122]
Main component, TQ	20 and 40 mg/kg, i.p.	Guinea pigs	Showed antitussive effect	[123]
Hydroethanolic	0.08 g daily	Sulfur mustard exposed guinea pigs	Reduced tracheal responsiveness to methacholine and also decreased total and differential WBC count	[124]
Hydroethanolic	0.08 g daily	Sulfur mustard exposed guinea pigs	Decreased tracheal responsiveness and lung inflammation including, percentage of eosinophil and monocyte, neutrophil, and lymphocyte number	[118]
*N. sativa* seeds	2 g	Asthmatics and nonasthmatics subjects	Increased FVC in asthmatics' group and PEFR in nonasthmatics' group	[125]
Capsules	40–80 mg/kg/day, p.o.	Rhinitis, atopic eczema and asthma	Reduced plasma and urine levels of IgE, eosinophil count, and endogenous cortisol	[126]
Supplement	1 and 2 g/day	Asthma patients	Increased PFT values, including FEF25–75% and FEV1 %, improved PEF, decreased FeNO and serum IgE, and increased serum IFN-gamma	[127]
*N. sativa* seeds	0.1–3.0 mg/ml	Rabbit jejunum, guinea pig trachea smooth muscle	Induced relaxant effects on carbachol-, histamine- or K(+)-induced contractions in guinea-pig trachea smooth muscle trough calcium channel blocking effects	[128]
Aqueous extract	0.25, 0.5 and 1 g%	Guinea pig tracheal smooth muscle	Showed calcium antagonist effects	[129]
Aqueous extract	0.25, 0.5 and 1 g%	Guinea pigs isolated trachea chain	Showed relaxant effect by stimulatory effect on *β*2- adrenoceptors	[130]
Aqueous extract	0.8, 1.2, 1.6 and 2.0 g%	Guinea pig tracheal chains	Showed significant relaxant effects	[131]
Hydroethanolic extract	0.125 and 0.250 mg/ml	Guinea pig tracheal chains	Decreased tracheal response to methacholine and ovalbumin, improved WBC cell and eosinophil counts in the lung lavage fluid	[132]
Methanolic fractions	20%, 40%, 60%, 80%, and 100%	Guinea pig tracheal chains	Showed significant relaxant effects	[133]
Aqueous extract	2–14 mg/mL	Contractile responses of rat isolated aorta	Showed vasodilator effect	[134]
Boiled extract	50 and 100 mg/kg	Asthmatic patients	Increased all measured PFTs values, bronchodilatory effect	[135]

IL: interleukin; IFN-*γ*: interferon-gamma; TNF-*α*: tumor necrosis factor-*α*; NO: nitrogen oxide; Ig: immunoglobulin; BAL: bronchoalveolar lavage; PEFR: peak expiratory flow rate; PFT: pulmonary function tests; FVC: forced vital capacity.

## Data Availability

No data were used to support this study.

## Abbreviations

Ach: Acetylcholine
ACT: Asthma control test
BAL: Bronchoalveolar lavage
COPD: Chronic obstructive pulmonary disease
DCs: Dendritic cells
ET: Endothelin
FEF: Forced expiratory flow
FEV1: Forced expiratory volume in 1 second
FOXP3: Forkhead box P3
FVC: Forced vital capacity
IBD: Inflammatory bowel diseases
ICAM: Intracellular cell adhesion molecular
IFN*γ*: Interferon-gamma
IgE: Immunoglobulin E
IL: Interleukin
iNOS: Induced nitric oxide synthase
MAO-B: Monoamine oxidase B
MCP-1: Chemoattractant protein 1
MMP-2: Mediated matrix metalloproteinase 2
NO: Nitric oxide
OVA: Ovalbumin
PBMC: Peripheral blood mononuclear cells
PEFR: Peak expiratory flow rate
PFT: Pulmonary function tests
PLA2: Phospholipase A2
POP: Polysaccharide
ROS: Reactive oxygen species
SI: Stimulation indices
SM: Sulfur mustard
SNP: Sodium nitroprusside
Th: T-helper
TLR-4: Toll-like receptor 4
TNF*α*: Tumor necrosis factor-*α*
TQ: Thymoquinone
VCAM: Vascular cell adhesion molecule
WBC: White blood cells
WHO: World Health Organization
